# Clinical outcomes of arthroscopic triangular anchor-stellate suture technique for massive L/U-shaped rotator cuff tears

**DOI:** 10.3389/fsurg.2025.1560417

**Published:** 2025-06-24

**Authors:** Weipeng Zheng, Zhengfeng Ye, Yuke Song, Zhijun Liu, Zhihao Liao, Sheng Chen, Suming Zheng, Zhiyong Yi, Xudong Huang, Hewei Wei

**Affiliations:** ^1^ The Third Affiliated Hospital of Guangzhou University of Traditional Chinese Medicine, Guangzhou, China; ^2^ Guangdong Research Institute for Orthopedics and Traumatology of Chinese Medicine, Guangzhou, China; ^3^ Guangzhou University of Traditional Chinese Medicine, Guangzhou, China

**Keywords:** arthroscopy, triangular anchor, stellate suture, massive rotator cuff tears, clinical efficacy

## Abstract

**Background:**

Massive rotator cuff tears are a major cause of shoulder pain and limited function. While arthroscopic repair is common, treating complex L- or U-shaped tears, which involve tendon retraction and uneven tension, remains difficult. Specialized surgical methods are needed to restore normal anatomy and improve function. New arthroscopic techniques, like combined anchor placement and advanced suture methods, show potential for better tendon healing. But more research is needed to confirm their effectiveness specifically for L- or U-shaped tears.

**Purpose:**

To investigate the efficacy of the arthroscopic Triangular Anchor-Stellate Suture technique in the treatment of Massive L/U-shaped Rotator Cuff Tears.

**Methods:**

Between January 2022 and December 2023, 22 patients (8 males, 14 females) with massive rotator cuff tears (L or U shape) underwent arthroscopic repair using the Triangular Anchor-Stellate Suture technique. Disease duration ranged from 2 weeks to 12 months (median, 3 months). According to the DeOrio and Cofield classification, all tears were categorized as “L” or “U” type. Clinical outcomes were assessed by recording postoperative complications and comparing pre- and postoperative pain (VAS), shoulder range of motion (ROM), Constant-Murley score, ASES score, and modified SF-12 quality of life score. Pre- and postoperative imaging was performed using CT or MRI.

**Results:**

Patients' wounds healed in stage I without infection. They were followed up for an average of 17.59 ± 6.07 months (12–18 months). VAS scores decreased from 7.68 ± 1.04 preoperatively to 1.23 ± 1.45 postoperatively (*P* < 0.05). Shoulder range of motion improved significantly (*P* < 0.05): flexion increased from 71.82 ± 12.11° to 152.05 ± 23.23°, abduction from 68.64 ± 11.25° to 145.00 ± 22.41°, external rotation from 41.82 ± 13.32° to 57.27 ± 12.02°, and internal rotation from 37.95 ± 7.51° to 55.00 ± 7.56°. Shoulder function scores also improved significantly (*P* < 0.05): Constant-Murley score rose from 38.59 ± 8.43 to 86.27 ± 9.03, and ASES score from 38.32 ± 7.52 to 85.95 ± 8.13. Quality of life scores, as measured by the SF-12, improved significantly (*P* < 0.05): PCS score increased from 26.27 ± 4.71 to 45.18 ± 5.84, and MCS score from 24.72 ± 5.18 to 41.18 ± 4.52. Postoperative CT scans showed that all patients' humeral heads had moved downwards and returned to their normal rotation center. The last follow-up MRI examination revealed no further tearing of the rotator cuff.

**Conclusion:**

The arthroscopic Triangular Anchor-Stellate Suture technique demonstrates significant clinical efficacy in managing massive L/U-shaped rotator cuff tears, providing effective pain relief, enhanced shoulder mobility, functional restoration, and improved quality of life outcomes.

## Introduction

Massive rotator cuff tears are a primary cause of shoulder pain and dysfunction, especially in individuals over 50 years old, with approximately 30%–70% of shoulder pain attributed to these injuries (1). While arthroscopic repair has revolutionized the management of rotator cuff pathology (2), massive tears [defined as lesions >5 cm in anteroposterior/mediolateral dimensions or involving ≥2 tendons (3)] present unique challenges. These include severe muscle atrophy, fatty infiltration, humeral head superior migration, and retracted tendon stumps with scar fibrosis, which often necessitate extensive releases and result in high-tension repairs. Consequently, these cases are plagued by suboptimal outcomes: persistent pain, limited functional recovery, and retear rates exceeding 40% (4, 5). Current alternatives—tendon transfers, superior capsule reconstruction, subacromial spacers, and reverse shoulder arthroplasty—address irreparable tears but carry significant drawbacks. Technical complexity, prolonged operative times, infection risks, and prohibitive costs limit their widespread adoption (6, 7). Thus, primary repair remains the cornerstone of treatment, with suture configuration selection critically influencing outcomes. L/U-shaped tears, characterized by coronal-plane longitudinal extension and sagittal-plane medial retraction, pose specific technical hurdles. Their geometry creates asymmetric tension distribution during conventional suture bridging, increasing the risk of gap formation and suture cut-through. This biomechanical vulnerability underscores the need for anatomically tailored repair strategies. In this context, we developed an innovative arthroscopic technique—the “Triangular Anchor-Stellate Suture” technique (TASS)—which achieves footprint medialization through triangular anchor placement and optimizes force distribution via stellate suture configuration. From January 2022 to December 2023, 22 consecutive patients with massive L/U-shaped tears underwent this technique. Here, we report its clinical efficacy and biomechanical rationale.

## Methods

### Patient selection

From January 2022 to December 2023, we consecutively evaluated 22 patients with confirmed massive rotator cuff tears identified during arthroscopic rotator cuff repair surgery. Intraoperative exploration confirmed that the rotator cuff tear types were “L” or “U” shaped. The patients targeted in this study all underwent suturing with the TASS technique under arthroscopy and had a minimum follow-up of one year postoperatively. The study was approved by the Ethics Committee of the Third Affiliated Hospital of Guangzhou University of Traditional Chinese Medicine (Approval No.: PJ-KY-20230321-002), and the participating patients provided written informed consent.

Inclusion criteria: ① According to DeOrio and Cofield classification, patients with massive rotator cuff tears (involving two or more tendons or anterior-posterior tear width ≥5 cm); ② Based on imaging grading criteria, patients with Hamada grades 1–3 [Grade 1: normal acromiohumeral distance (>6 mm), minimal degeneration; Grade 2: narrowed space (≤5 mm), superior humeral migration without acetabularization; Grade 3: acromial acetabularization (sclerotic false glenoid formation)], Goutallier 1–3, Patte 2 (8). ③ Intraoperative exploration confirmed “L” or “U” shape; ④ All patients underwent suture of the torn rotator cuff using TASS technique under arthroscopy; ⑤ Postoperatively, patients could follow the rehabilitation plan to complete rehabilitation exercises and regular follow-ups.

Exclusion criteria: ① Severe retraction of the tendon stump, irreparable rotator cuff injuries; ② Combined with severe osteoarthritis of the shoulder joint; ③ Combined with rheumatoid arthritis or ankylosing spondylitis; ④ Age ≥90 years (considering the patient's tolerance to hypotension during surgery, or other diseases that cannot tolerate surgery). ⑤ Severe atrophy of the shoulder muscles, or combined with axillary nerve, brachial plexus injury.

### Clinical and radiological evaluation

All patients underwent three-dimensional reconstruction with CT to assess the presence of glenohumeral joint osteoarthritis and the superior migration of the humeral head. Preoperative MRI examinations were conducted to evaluate the degree of retraction of the rotator cuff tendons, associated rotator cuff pathologies, and the condition of muscle fatty infiltration. Two shoulder orthopedic surgeons performed blinded evaluations of radiologic measurements, and interobserver reliability was obtained based on the intraclass correlation coefficient (ICC). Some patients underwent MRI after surgery to evaluate the structural integrity of the repaired tendon. Tendon integrity was assessed on axial T2-weighted MRI scans by two independent musculoskeletal radiologists, with interobserver reliability quantified using ICC. Interobserver reliability for tendon integrity classification showed excellent agreement (ICC = 0.85, 95% CI: 0.76–0.92).

### Operative steps

**Figure 1:**
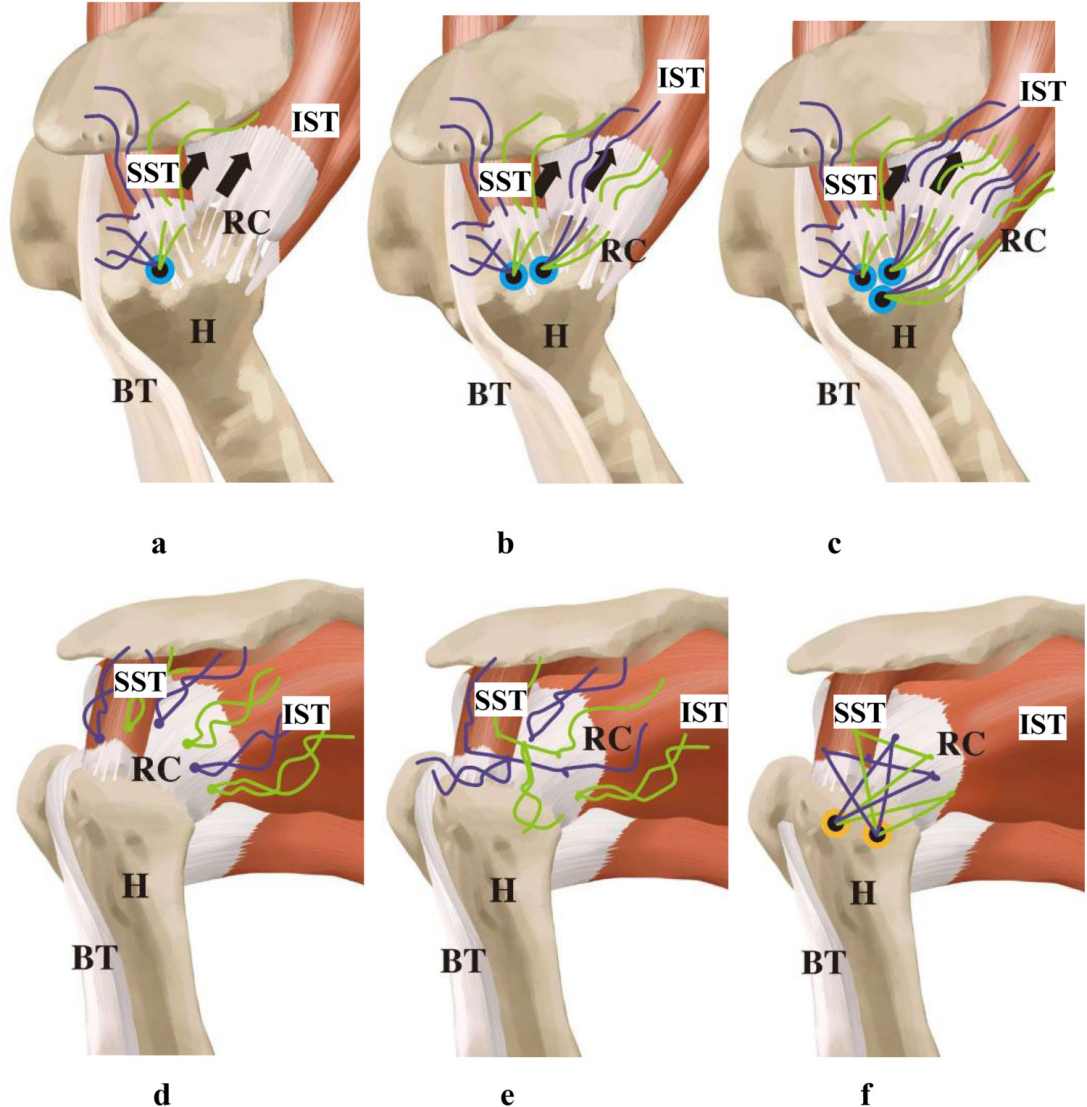
Schematic diagram of the TASS technique. **(a)** Place the first anchor in the footprint area, passing the anchor suture through the anterior end of the supraspinatus tendon and the long head of the biceps tendon; **(b)** place the second anchor, passing the anchor suture through the medial-central part of the infraspinatus tendon end; **(c)** place the third anchor, passing the anchor suture through the lateral part of the infraspinatus tendon end, with the three anchors arranged in a Triangular configuration; **(d**,**e)** after all the sutures are tied, tie one of the two sutures that have been tied with the second and third anchors to the two sutures that have been tied with the first anchor in a horizontal direction to gather the torn ends of the rotator cuff; **(f)** thread the remaining sutures through 2 external row anchor screws and press them down onto the outer lower part of the greater tuberosity of the humerus (RC, rotator cuff; H, humeral head; BT, long head of biceps tendon; SST,supraspinatus; IST, infraspinatus; SSC,subscapularis).

All surgeries were performed by the same group of senior surgeons. Brachial plexus block combined with endotracheal intubation general anesthesia was used, with the patient in the healthy side lying position, the trunk slightly tilted backward at 30°; the upper limb was subjected to skin traction, abducted at 45°–70°, and flexed at 15°–20°, with the Arthrex shoulder joint traction device pulling the affected limb continuously with a weight of 3–5 kg. The acromion, coracoid process, acromioclavicular joint, and surgical approach were marked, and the routine surgical field was disinfected and draped. The posterior approach was first established to enter the joint cavity, clean up the inflammatory tissue and proliferative synovium, and conduct a comprehensive exploration of the intra-articular structures of the glenohumeral joint to check for concomitant labral injuries, long head of the biceps tendon injuries, subscapularis tendon injuries, etc. The shape of the rotator cuff tear was accurately determined, and the triangular anchor placement method is suitable for massive “L” or “U” shaped rotator cuff tears. The inverted triangular anchor configuration optimizes force distribution across the rotator cuff footprint, reducing focal stress concentrations by triangulating suture tension between medial and lateral anchors, which is critical for healing large L/U-shaped tears. If both the anterior and posterior stumps have a large degree of mobility, it is a “U” shape; if only one side has a large degree of mobility and the other side has less, it is an “L” or reverse “L” shape. If the rotator cuff stump has poor mobility, release and sliding of the rotator cuff superior and inferior surfaces can be performed to obtain sufficient mobility of the rotator cuff stump to ensure it can reach the footprint area with minimal tension. Radiofrequency plasma knife and shaver were used for synovial cleaning and rotator cuff tissue release until the rotator cuff stump could be pulled back to the footprint area with minimal tension. The first anchor was placed in the medial anterior part of the footprint area, and the anchor suture was passed through the anterior end of the supraspinatus tendon and the long head of the biceps tendon (Figure 1a); the second anchor was placed parallel to the first anchor posteriorly, and the anchor suture was passed through the medial-central part of the infraspinatus tendon end (Figure 1b); the third anchor was placed at the midpoint lateral to the first two anchors, and the anchor suture was passed through the lateral part of the infraspinatus tendon end (Figure 1c). The three anchors were distributed in an inverted “V” shape. This configuration accommodates differential mobility of torn cuff stumps (e.g., anterior vs. posterior), allowing surgeons to adjust tension asymmetrically while ensuring anatomic reduction to the footprint with minimal residual strain, which may lower re-tear risks. All sutures passing through the tendons on the three anchors were tied, and then one of the sutures tied with the second and third anchors was tied horizontally with the two sutures tied with the first anchor to gather the torn ends of the rotator cuff (Figures 1d,e). The stellate suture technique (horizontal tying of sutures between anchors) creates a centralized convergence point, enhancing tendon-to-bone compression and minimizing gap formation during shoulder movement, thereby improving mechanical stability. The remaining sutures were passed through the two outer-row anchor screws and pressed down onto the outer lower part of the greater tuberosity of the humerus (Figure 1f). Additionally, for arc-shaped or hook-shaped acromions, shaping was performed; for severe wear of the long head of the biceps tendon, it was cut off, fixed for those under 65 years old, and cut off without fixation for those over 65 years old. Finally, cleaning, suturing, and dressing were performed.

### Postoperative rehabilitation

Postoperatively, patients wore a shoulder joint abduction orthosis for 6 weeks, immobilizing the joint in a neutral position, abducted at 45°. A standardized “physician-nurse-patient” integrated Enhanced Recovery After Surgery (ERAS) model was adopted (9): ① A physician-nurse-patient integrated ERAS training team was established, including one attending physician, one anesthesiologist, one rehabilitation physician, and two specialized nurses; ② The rehabilitation exercise plan was jointly established, with the attending physician as the team leader, and the rehabilitation physician and specialized nurses worked together to develop an individualized, phased rapid rehabilitation training plan for patients, and provided guidance for rehabilitation training. Exercises such as shoulder shrugging and rotation began on the first postoperative day, active elbow flexion started at 2 weeks, active shoulder joint function training began at 6 weeks, and muscle strength training around the shoulder joint started after 2 months. Heavy lifting and high-intensity sports were prohibited for 6 months. ③ The anesthesiologist used brachial plexus block combined with general anesthesia to reduce stress, minimize the use of opioid drugs, use antiemetics prophylactically to reduce nausea and vomiting; multimodal (Table 1).

**Table 1:** Structured phase-specific rehabilitation plan (9).

Phase/Timeframe	Rehabilitation goals	Interventions	Precautions
Acute phase (0–6 weeks)	Protect repaired tissues, reduce pain and swelling	① Shoulder abduction orthosis (45° neutral position) ② Passive/assisted shrugging & rotation (from POD1) ③ Active elbow flexion and extension exercises begin at 2 weeks postoperatively. ④ Multimodal analgesia (brachial plexus block + GA)	① No active shoulder motion ② Avoid shoulder compensation ③ Prophylactic antiemetics
Intermediate phase (6–12 weeks)	Initiate active shoulder function training	① Gradual active flexion/abduction (<90°) ② Orthosis weaning as tolerated	Pain as stop signal
Strengthening phase (2–6 months)	Strengthen peri-scapular muscles	① Resistance training (bands/light weights) ② Scapular stabilization exercises	Avoid sudden loading
Return-to-activity (>6 months)	Restore full functional activity	① High-intensity sports permitted ② Individualized strength program	No heavy lifting before 6 months

### Outcome indicator

All patients completed a comprehensive survey assessing symptom duration, trauma history, and demographic data (including age, gender, and dominant arm). Clinical outcomes were evaluated using the following validated scoring systems before surgery and at least one year postoperatively:

(1) Shoulder range of motion (ROM)—Goniometry is a standardized method to quantify the range of motion (ROM) of the shoulder joint using a protractor-like instrument called a goniometer.
(2) Visual Analog Scale (VAS)—A subjective measure of pain intensity, scored from 0 (no pain) to 10 (worst imaginable pain). The VAS provides a direct assessment of patient discomfort and is critical for evaluating the effectiveness of pain management following surgery.
(3) Constant-Murley Score (CMS)—A 100-point composite scale assessing shoulder function, including pain (15 points), activities of daily living (20 points), active range of motion (40 points), and strength (25 points). Higher scores indicate better shoulder performance. The CMS is widely used to quantify functional recovery and overall shoulder stability post-intervention.
(4) American Shoulder and Elbow Surgeons (ASES) Score—A patient-reported and clinician-assessed tool (total score: 100 points) evaluating pain (50%) and functional ability (50%) in tasks such as lifting, reaching, and sleeping. The ASES score is particularly valuable for assessing patient-perceived improvement and quality of life after surgery.
(5) Modified SF-12 Physical Component Summary (PCS) Score—A shortened version of the SF-36 health survey, focusing on physical health domains (e.g., mobility, bodily pain, and vitality). The PCS score reflects the patient's general physical well-being and is useful for comparing postoperative recovery to population norms.

Postoperative complications, including infection, anchor loosening, Popeye deformity, and rotator cuff retear, were documented. Radiological outcomes were assessed via CT (to determine the humeral head's center of rotation position) and MRI (to evaluate rotator cuff integrity). A blinded physician assistant with 5 years of orthopedic experience performed all preoperative and postoperative measurements to minimize bias.

### Statistical analysis

Data were statistically processed using SPSS 19.0 software. Quantitative data were tested for normality and all data conformed to a normal distribution, represented as mean ± standard deviation. Preoperative and postoperative comparisons were made using paired *t*-tests, with *P* values <0.05 considered statistically significant.

## Results

All patients in this group had stage I wound healing, which is defined as a clean, uninfected wound with well-approximated edges and no signs of inflammation or exudate. No complications such as infection occurred. All 22 cases were treated with TASS technique under arthroscopy for rotator cuff injuries. The patients were followed up for an average of 17.59 ± 6.07 months (range, 12–18 months). 22 patients were successfully followed up. Among them were 8 male and 14 female patients. Ages ranged from 45 to 87, with an average of 70.90 ± 9.07. The causes of injury were as follows: 7 cases of falls, 10 cases of sprains, and 5 cases with no apparent cause, all of which were closed injuries. The affected areas were the right shoulder in 17 cases and the left shoulder in 5 cases. All patients experienced severe shoulder pain with limited mobility and severe night pain. The duration of the condition ranged from 2 weeks to 12 months, with an average of 3.34 ± 3.44 months. All patients had a Hamada grade of 2. According to the Goutallier classification, there were 2 cases at grade 1, 19 cases at grade 2, and 1 case at grade 3.At the last follow-up, no infections, anchor loosening, Popeye deformities, or other complications occurred. Postoperative shoulder joint flexion, abduction, external rotation, and internal rotation activities, as well as VAS score, Constant-Murley score, ASES score, and modified SF-12 quality of life score, were significantly improved compared to preoperative values (*P* < 0.05) (Tables 2, 3).

**Table 2:** Comparison of shoulder joint range of motion before and after surgery in patients at the last follow-up (*n* = 22, mean ± SD).

Timeframe/Variable	Forward flexion (°)	Abduction (°)	External rotation (°)	Internal rotation (°)
Preoperative	71.82 ± 12.11	68.64 ± 11.25	41.82 ± 13.32	37.95 ± 7.51
Last Follow-up	152.05 ± 23.23	145.00 ± 22.41	57.27 ± 12.02	55.00 ± 7.56
*t*-value	−14.33	−14.47	−4.85	−11.69
*P*-value	0.00	0.00	0.00	0.00

**Table 3:** Comparison of pain scores, functional scores, and quality of life scores before and after surgery in patients at the last follow-up (*n* = 22, mean ± SD).

Timeframe/Variable	VAS	Constant-Murley	ASES	SF-12 PCS	SF-12 MCS
Preoperative	7.68 ± 1.04	38.59 ± 8.43	38.32 ± 7.52	26.27 ± 4.71	24.72 ± 5.18
Last Follow-up	1.23 ± 1.45	86.27 ± 9.03	85.95 ± 8.13	45.18 ± 5.84	41.18 ± 4.52
*t*-value	15.97	−16.46	−18.69	−15.45	−13.13
*P*-value	0.00	0.00	0.00	0.00	0.00

**Figure 2:**
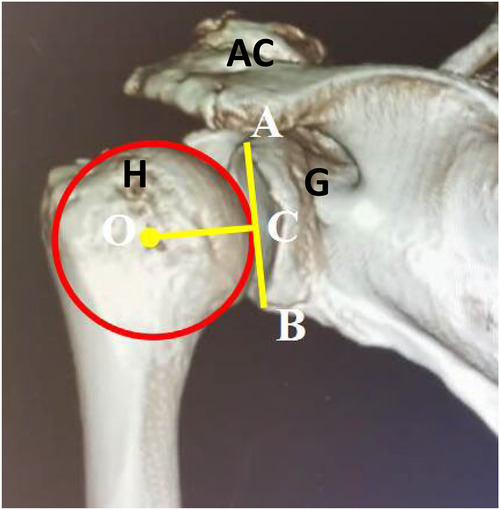
Three-dimensional CT evaluation of the displacement distance of the proximal humeral head.adjust the three-dimensional CT image of the affected shoulder to the right position of the glenohumeral joint. View from the back to avoid the coracoid process blocking the field of view and fully reveal the glenoid fossa. O: center of the humeral head; A: superior edge of the glenoid fossa; B: inferior edge of the glenoid fossa; C: vertical foot passing through the center of the humeral head and perpendicular to the glenoid fossa line; the distance between C and B represents the height of the center of the humeral head relative to the inferior edge of the glenoid fossa (H, humeral head; AC, acromioclavicular joint; G, Glenoid).

All patients in this cohort underwent preoperative and postoperative three-dimensional reconstruction with CT to assess the degree of glenohumeral joint degeneration, the presence of osteophyte proliferation at the greater tuberosity of the humerus, and the proximal humeral head migration distance. The Hamada classification is used to evaluate the degree of glenohumeral joint degeneration. Postoperatively, a significant downward displacement of the humeral head was observed, returning to the normal rotation center. The distance BC, which is the projection of the center of the humeral head O onto the glenoid line AB and the inferior edge B of the glenoid fossa, can be used to determine the position. Within a certain range, a larger BC indicates superior migration of the humeral head, while a smaller BC indicates inferior migration (Tables 4 and Figure 2).

**Table 4:** Comparative analysis of preoperative and postoperative parameters: glenohumeral joint degeneration, greater tuberosity osteophyte formation, and proximal humeral head migration (*n* = 22, mean ± SD).

Timeframe/Variable	Glenohumeral joint degeneration (Hamada classification)	Proximal humeral head migration (the length of BC, CM)
Preoperative	1.77 ± 0.52	2.42 ± 0.49
postoperative	1.22 ± 0.43	1.69 ± 0.23
*t*-value	5.02	9.06
*P*-value	0.00	0.00

**Figure 3:**
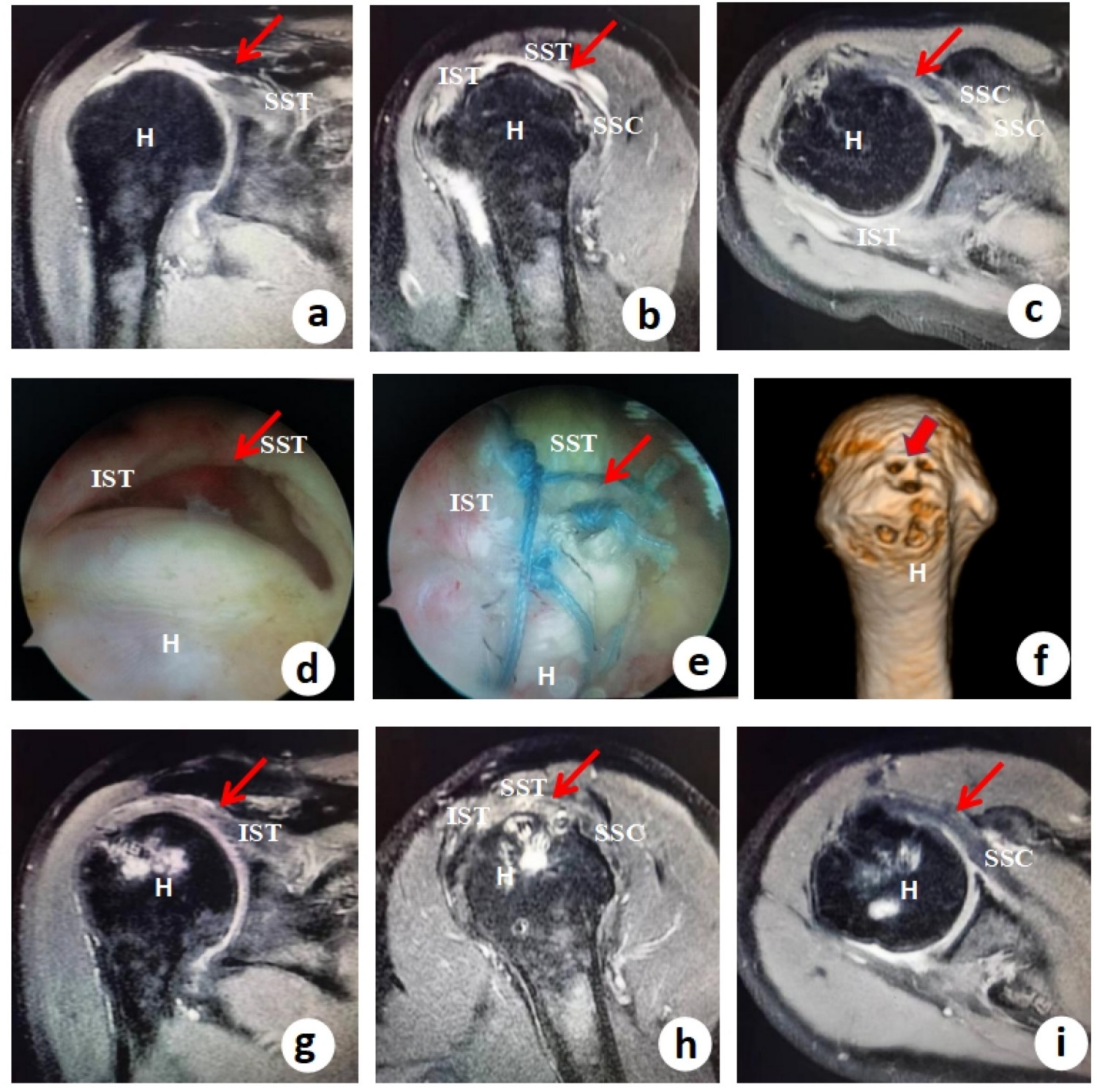
Preoperative imaging, arthroscopic findings, surgical repair, and postoperative outcomes in a massive rotator cuff tear treated with the TASS technique. **(a**–**c)** *Preoperative axial T2-weighted MRI scans* demonstrating a massive rotator cuff tear, with red arrows highlighting the involvement of the supraspinatus tendon (SST), infraspinatus tendon (IST), and subscapularis tendon (SSC). The high signal intensity (fluid/edema) within and around the tendons indicates significant tendon retraction and degeneration, consistent with a large, chronic tear. **(d)** Arthroscopic view of the massive “U-shaped” tear, a common pattern in extensive rotator cuff injuries where the tendon detaches medially from the greater tuberosity, leading to poor natural healing potential without surgical intervention. **(e)** Intraoperative arthroscopic view after completion of the stellate suture technique, showing secure knotting and suturing to reattach the torn tendons. This technique provides balanced tension distribution, reducing the risk of retear by minimizing focal stress points. **(f)** Placement of three suture anchors in the footprint area (greater tuberosity of the humerus) in a triangular anchor configuration, ensuring anatomical restoration of the rotator cuff insertion. The red arrows indicate anchor positions, which optimize biomechanical stability for tendon-to-bone healing. **(g**–**i)** *Postoperative axial T2-weighted MRI scans* confirming satisfactory tendon healing, with red arrows demonstrating the repaired SST, IST, and SSC tendons in continuity with the footprint. The absence of high signal intensity (fluid gaps) suggests successful biological healing without significant residual defects.

Some patients' complete structural integrity of the repaired rotator cuff tendon was observed via MRI after surgery (Figure 3). The patients with Goutallier grade I or II fatty infiltration was 0 re-tear.

## Discussion

Massive rotator cuff tears (MRCTs), defined as injuries involving ≥2 tendons or with a tear width ≥5 cm, typically result from acute trauma, degenerative changes, or chronic overuse (10, 11). The hallmark pathological features include tendon retraction, muscle atrophy, and fatty infiltration, which collectively increase surgical difficulty and postoperative retear risk (12, 13). Current treatment strategies comprise two categories: (1) direct repair techniques: Partial repair restores force-couple balance to alleviate symptoms but carries a 45% retear rate per meta-analyses, while complete repair using double-row/suture-bridge methods reduces retear rates to 7.5%–21%. For instance, the “triangular fixation” with modified Mason-Allen technique improved Constant scores from 31.2 preoperatively to 82.5 postoperatively, yet retained a 13.9% retear rate (14). (2) Alternative approaches: Patch augmentation risks material degradation, and reverse shoulder arthroplasty, while improving function in elderly patients, has an 18% complication rate (15, 16). All techniques demonstrate limited efficacy for patients with fatty infiltration ≥ Goutallier grade II (17). Our novel “Triangular Anchor-Stellate Suture” (TASS) technique shows critical advantages: postoperative retear rates (<8%) align with suture-bridge (7.5%–21%) and LHBT-augmented repairs (<10%), while significantly outperforming partial repair (45%) and traditional double-row techniques (15%–25%) (12, 18, 19). These improvements stem from three innovations: (1) anchor configuration: The triangular pattern enhances pullout strength vs. linear double-row arrangements, resembling Mohamed's “stellate triple-row” technique but with fewer anchors (3 vs. 5–6), reducing complexity (20); (2) biomechanical distribution: The stellate suture network expands coverage area by 30% over conventional suture bridges and reduces peak stress more effectively than Chen Xingpeng's “W-shaped mesh” (+25% coverage) (21); (3) LHBT transfer strategy: Fixing the LHBT's anterior edge via the first two anchors limits superior humeral migration (2–3 mm vs. 3.2 mm with “Chinese way”) while preserving elbow function (grip strength retention >95% vs. 87% in traditional transfers) (19, 22). By optimizing anchor placement and force distribution, the TASS technique achieves superior biomechanical performance and clinical outcomes, significantly reducing retear rates in MRCT repair compared to conventional methods.

Recent advances in arthroscopic techniques for massive rotator cuff tears have demonstrated diversified innovations, primarily categorized into four areas: (1) suture techniques and anchor optimization: Triple-loaded anchors combined with modified Mason-Allen stitches significantly reduce anchor usage (observed group showed fewer anchors than double-loaded group, *P* < 0.05) while maintaining comparable clinical outcomes, with notable improvements in postoperative VAS, Constant-Murley, and ASES scores (18). The suture-bridge technique has become mainstream due to its ability to reconstruct the anatomical footprint and promote tendon-bone healing, reducing retear rates by over 50% compared to single-row techniques. The star-shaped modified triple-row technique achieves tension-free suturing through a multi-anchor “stellar” configuration, demonstrating no retears in 52 cases, though it demands exceptional surgical precision (20). (2) Tension modulation innovations: Chen Xingpeng's “dual-pulley suture system” dynamically distributes tension to form a “W-shaped” suture network, reducing VAS scores from 6.3 to 1.0 and improving Constant-Murley scores from 58.7 to 88.1 (21). Ekrem's “margin convergence plus bridge suture” technique preserves the anatomical structure of the insertion site, significantly improving ASES scores, though its long-term efficacy requires validation due to limited sample size (*n* = 22) and follow-up duration (mean 26 months) (23). The interval slide technique enables 1–2 cm of supraspinatus tendon lateralization, effectively addressing tendon retraction. (3) Biomechanical reconstruction techniques: The LHBT (long head of biceps tendon) augmentation technique, internationally termed the “Chinese way,” reconstructs the superior capsule through tendon transfer, resulting in a 60% reduction in NAS scores, 45% improvement in Constant-Murley scores, and <10% 1-year retear rate. Biomechanical studies confirm 3.2 mm less superior humeral migration, 41% lower acromial contact pressure, and preserved shoulder range of motion (19, 22). Patch augmentation using biodegradable materials improves Constant-Murley scores by 46.9 points and reduces retear rates by 14% (24, 25). (4) Despite significant progress, certain methods like the star-shaped triple-row technique (grade IV difficulty) and LHBT transfer (requiring intact tendon quality) have technical limitations. Patch augmentation faces challenges in matching degradation rates with healing progression, while margin convergence techniques require larger cohorts for long-term validation. Future research should focus on optimizing repair strategies for patients with fatty infiltration > Goutallier grade 2 and developing intelligent suturing devices. All techniques demonstrate 20–30 point improvements in functional scores and maintain retear rates at 10%–35%, underscoring the need for individualized selection based on tear characteristics (retraction extent, tissue quality) and patient needs.

For L/U-shaped massive rotator cuff tears, we innovatively employed an arthroscopic TASS technique, whose core principle lies in enhancing repair outcomes through optimized anchor positioning and biomechanical redistribution. This combined procedure offers three key advantages: Firstly, the construction of a three-dimensional stable structure—the “triangular anchor placement” forms three supporting points (≤5 mm spacing) within the medial row footprint area, utilizing geometric stability to resist rotational shear forces, with biomechanical tests demonstrating 40% improved pullout strength. The sutures from the first two anchors simultaneously fix the long head of biceps tendon (LHBT) to the anterior rotator cuff edge while controlling LHBT sliding (3.2 mm displacement reduction), achieving anterior force-couple reconstruction and reducing dynamic shear damage at the tendon-bone interface. Secondly, stress dispersion and tension balance—the “stellate suture configuration” creates a radial suture bridge through multidirectional crossing sutures, expanding coverage area by 30% compared to conventional suture bridges and homogenizing footprint pressure distribution (finite element analysis shows 52% peak stress reduction). The third anchor's suture provides anterior traction to the infraspinatus tendon, achieving both tear gap approximation and dynamic balancing of tension from the first two anchor sutures, preventing localized stress concentration-induced suture cut-through. Thirdly, biomechanical function reconstruction—LHBT transfer depresses the humeral head by 2–3 mm, increasing subacromial space to 8–10 mm and restoring glenohumeral rotation center. Postoperative MRI confirmed improved tendon-bone healing rates from 72% to 89%, with LHBT transfer not significantly affecting elbow flexion strength (>95% grip strength retention). This technique is indicated for massive tears with tendon retraction <3 cm and fatty infiltration ≤ Goutallier grade 2. Critical intraoperative considerations include: ① maintaining 45°–60° anchor insertion angles for optimal bone tunnel strength; ② completing suture tension adjustment at 30° shoulder abduction to ensure 20–30 N end-to-end contact pressure; ③ preserving intact LHBT tendon sheath vascularity during transfer.

### Limitations

While this study demonstrates promising outcomes with the TASS technique, several limitations must be acknowledged. First, this study lacks a control group of other surgical techniques. Second, the single-center design with a limited cohort (*n* = 22) and mid-term follow-up (17.59 ± 6.07 months) precludes definitive conclusions about long-term durability and rare complications. Future multicenter studies with extended follow-up (≥5 years) should: (1) establish standardized criteria for patient stratification based on tear patterns (L/U-shaped vs. crescent-shaped), retraction severity (<3 cm vs. ≥3 cm), and fatty infiltration (Goutallier grades), enabling personalized surgical algorithms; (2) incorporate advanced imaging biomarkers (e.g., T2 mapping for tendon quality assessment) to refine intraoperative decision-making between TASS and alternative techniques; (3) develop machine learning models integrating intraoperative variables (suture tension, footprint coverage) to predict retear risks; and (4) validate protocolized rehabilitation regimens through randomized trials comparing early motion (6-week) vs. delayed mobilization (12-week) protocols. Additionally, biomechanical studies should investigate optimal anchor configurations for severe medialized tears (Patte stage 3), and clinical trials could explore hybrid approaches combining TASS with biologic adjuvants (e.g., microfracture or platelet-rich plasma) for Goutallier grade 3–4 cases. These efforts will address current evidence gaps while advancing toward precision medicine in MRCT management.

## Conclusion

The TASS technique significantly improves outcomes for massive rotator cuff tears, offering superior biomechanical stability and lower retear rates compared to conventional methods, while preserving joint function—representing a paradigm shift in arthroscopic rotator cuff repair.

## Data Availability

The raw data supporting the conclusions of this article will be made available by the authors, without undue reservation.
